# The Meeting Centre Support Programme: International Evaluation of a Dyadic Intervention in Dementia

**DOI:** 10.1093/geroni/igaa057.2735

**Published:** 2020-12-16

**Authors:** Dorota Szcześniak, Katarzyna Lion, Franka Meiland, Dawn Brooker, Elisabetta Farina, Rabih Chattat, Rose-Marie Dröes, Joanna Rymaszewska

**Affiliations:** 1 Wroclaw Medicla University, Wrocław, Dolnoslaskie, Poland; 2 Menzies Health Institute Queensland Griffith University, Nathan, Queensland, Australia; 3 University Medical Centers, VU University Medical Center, Amsterdam, Noord-Holland, Netherlands; 4 Association for Dementia Studies, University of Worcester, Worcester, England, United Kingdom; 5 IRCCS Fondazione Don Carlo Gnocchi, Milan, Lombardia, Italy; 6 University of Bologna, Bologna, Emilia-Romagna, Italy; 7 Amsterdam Public Health research institute, Amsterdam, Noord-Holland, Netherlands; 8 Wroclaw Medical University, Wrocław, Dolnoslaskie, Poland

## Abstract

In Europe, 10 million people are living with dementia. Most of them reside in their own home, cared for by their loved ones. As a consequence, there is a great need to provide both, people with dementia and their carers, tailored support. The Dutch Meeting Centres Support Programme (MCSP), adaptively implemented in three European countries within the JPND-MEETINGDEM project, is an excellent example of an effective dyadic psychosocial intervention, which seems to have no cultural barriers. The mixed-methods analysis showed that participant-dyads reported great satisfaction with MCSP. People with dementia experienced improvement of their quality of life, motivation and ability to participate in everyday activities, as well as improvement in their relationship with family members. Carers felt less burdened and highly appreciated the emotional and social support they received. Repeated user evaluation shows that this dyadic support effectively helps people with dementia and their families better deal with dementia.

